# Osteosarcoma of the spine: surgical treatment and outcomes

**DOI:** 10.1186/1477-7819-11-89

**Published:** 2013-04-18

**Authors:** Dapeng Feng, Xinghai Yang, Tielong Liu, Jianru Xiao, Zhipeng Wu, Quan Huang, Junming Ma, Wending Huang, Wei Zheng, Zhiming Cui, Huazi Xu, Yong Teng

**Affiliations:** 1 Spine Center, Changzheng Hospital, Second Military Medical University, 415 Fengyang Road, Shanghai, 200003, China; 2 Department of Orthopedic Surgery, Nantong First People’s Hospital, Nantong, 226001, China; 3 Department of Orthopedic Surgery, Second Affiliated Hospital of Wenzhou Medical College, Wenzhou, 325027, China; 4 Department of Orthopedic Surgery, Wulumuqi General Hospital of Lanzhou Military Command, People’s Liberation Army, Wulumuqi, 830000, China

**Keywords:** Osteosarcoma, Spine, Total *en bloc* spondylectomy, Total piecemeal spondylectomy

## Abstract

**Background:**

The goal of this study was to determine whether there are correlations between various options of surgical treatment and long-term outcome for spinal osteosarcoma.

**Methods:**

This was a retrospective review of 16 patients with spinal osteosarcoma, who underwent surgical treatment from 1999 to 2010. Seven patients were given total *en bloc* spondylectomy (TES), while nine received piecemeal resection (there were seven cases of total piecemeal spondylectomy, one of sagittal resection, and one of vertebrectomy). The outcome and prognosis of the patients were evaluated, grouped by surgical treatment.

**Results:**

All 16 cases were followed for an average of 42.4 months. At follow-up, all patients noted that pain had eased or had gradually disappeared. Three months after surgery, eight patients (50.0%) had improved 1 to 2 grades in their neurological status, based on Frankel scoring. Six (37.5%) patients experienced local recurrence of the tumor, nine (56.3%) had metastases, and five (31.3%) died of the disease. Of the six patients who received a wide or marginal *en bloc* resection, none developed local recurrence or died from the disease. Conversely, of the ten patients who received intralesional or contaminated resections, six (60%) relapsed and five (50%) died from the disease.

**Conclusions:**

TES, with a wide margin, should be planned for patients with osteosarcoma of the cervical and thoracolumbar spine, whenever possible. When the patients are not candidates for *en bloc* resection, total piecemeal spondylectomy is an appropriate choice for osteosarcoma in the mobile spine.

## Background

Osteosarcoma is one of the most common primary malignant tumors of the bone, arising frequently in the extremities and only rarely in the spine. It is a high-grade, malignant tumor with a poor prognosis. Various treatment methods have been advocated for osteosarcoma, including chemotherapy, radiation therapy, and surgical resection. Because osteosarcoma is locally aggressive, inadequate excision leads to a high rate of local recurrence and potential metastasis. Thus, a wide resection is generally agreed to be the optimal treatment option. The addition of neoadjuvant chemotherapy has further been shown to improve survival in patients with extremity osteosarcoma.

With the recent development of new resection and reconstruction techniques [1,2], *en bloc* resection can now be performed in selected patients with osteosarcoma of the spine [3]. Schwab *et al.*[4] reported a study involving 17 patients with osteosarcoma of the spine, in which 9 patients were candidates for and underwent *en bloc* spondylectomy. The authors concluded that *en bloc* resection is beneficial for survival. Because of complicated anatomical constraints, however, this procedure is not a feasible option for some patients. Furthermore, the association between patient outcome and various treatments for osteosarcoma of the spine is not known. Herein, we describe a retrospective study that evaluated the long-term outcome of two different surgical modalities, combined with chemotherapy and radiation therapy, for 16 patients with primary osteosarcoma of the spine.

## Methods

### Patient cohort

We retrospectively assessed 16 cases of patients with spinal osteosarcoma, who were enrolled in our center between September 1999 and January 2010. Ten patients were male and six patients were female. The age of the patients ranged from 15 to 58 at diagnosis, with an average age of 37.1 years. In total, six lesions were located in the cervical spine, six in the thoracic spine, and four in the lumbar spine. The most common symptom was chronic progressive pain, which was reported by 13 patients. Nine patients complained initially of severe pain, which required oral application of codeine or morphine to control. One patient had an injury history of the neck. The second most common presenting symptom was neurologic disorder, which was reported by 11 patients. All of the neurologic statuses were classified according to the Frankel score [5]: two patients were Grade B, three were Grade C, four were Grade D, and seven were Grade E. The period of symptoms between onset and diagnosis ranged from 0.5 months to 14 months (median: 4.3 months).

### Radiological studies

Appearance of the tumor by X-ray is informative for the diagnosis. Typically, patients with spinal osteosarcomas have a poorly defined lytic and sclerotic lesion involving the vertebra. In our study, radiologic findings were summarized and classified from existing radiology reports. Fourteen patients had osteoblastic, osteolytic, or mixed findings, and two patients had no diagnostic finding by X-ray. Pathologic fracture was observed in six cases.

Despite the utility of plain radiographs, computed tomography (CT) and magnetic resonance imaging (MRI) both offer improved resolution and better delineation of tumor extension. Using CT, we were able to confirm the presence of sclerotic, lytic, or mixed lesions with a thin, sclerotic border. MRI was informative, as tumor appearance in MRI depends heavily on the extent of ossification. Areas of non-ossification have relatively low signal intensity on T1-weighted images and a high signal on T2-weighted images, whereas areas of ossification appear dark in all sequences. In most of our cases, MRI revealed a mass with hypointense signal on T1WI and mixed hypointense and hyperintense signals on T2WI. No tumors involved the intervertebral space.

### Classification and staging

With classifying osteosarcomas, two systems are generally used: the Enneking system and the Weinstein-Boriani-Biagini (WBB) system. The Enneking system is based on the histologic grade of the tumor, the location of the lesion, and whether or not there are metastases [6]. When our tumors were classified using Enneking parameters, two cases were stage IIA, thirteen were stage IIB, and one was stage IIIA. While somewhat informative, the Enneking system was originally developed for tumors involving the extremities and has some limitations in the mobile spine so we also opted to classify our tumors using the WBB system.

**Figure 1:**
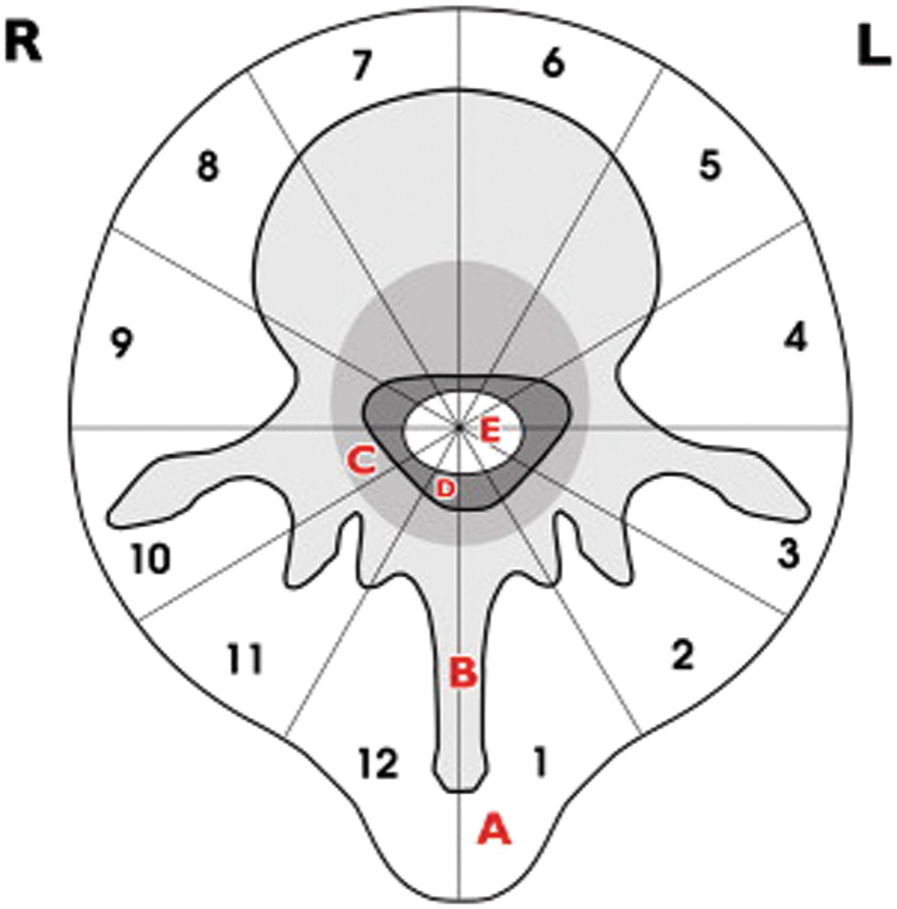
**Surgical staging of spinal tumors, according to the WBB system.** The transverse section is divided into 12 sectors (in a clockwise order) and into five concentric layers. (**A**) extraosseous soft tissue, (**B**) intraosseous (superficial), (**C**) intraosseous (deep), (**D**) extraosseous (extradural), and (**E**) extraosseous (intradural).

The WBB classification is often used to describe the anatomic site and the extension of the lesions in the mobile spine [7] (Figure 1). This system can help the surgeon to select optimal surgical treatment options. In our series, extraosseous paravertebral (layer A) involvement was found in 14 of 16 cases. For twelve cases, the tumor mass invaded the epidural space (layer D), whereas for five cases, the lesions were restricted to the anterior column (zones 4–9). In eleven cases, the lesions extended into both anterior and posterior columns. The tumor was limited to one vertebra in thirteen cases, two vertebrae in one case, and three vertebrae in two cases.

### Treatment: surgery and chemotherapy

Thirteen patients received a percutaneous CT-guided needle biopsy in our institution before surgical treatment was initiated. Two patients received an open biopsy with an immediate tumor excision, because of significant neurologic involvement. One patient (Case 13) received a CT-guided needle biopsy of his spinal tumor in another institution and came to our center after the pathologic examination revealed osteosarcoma.

**Figure 2:**
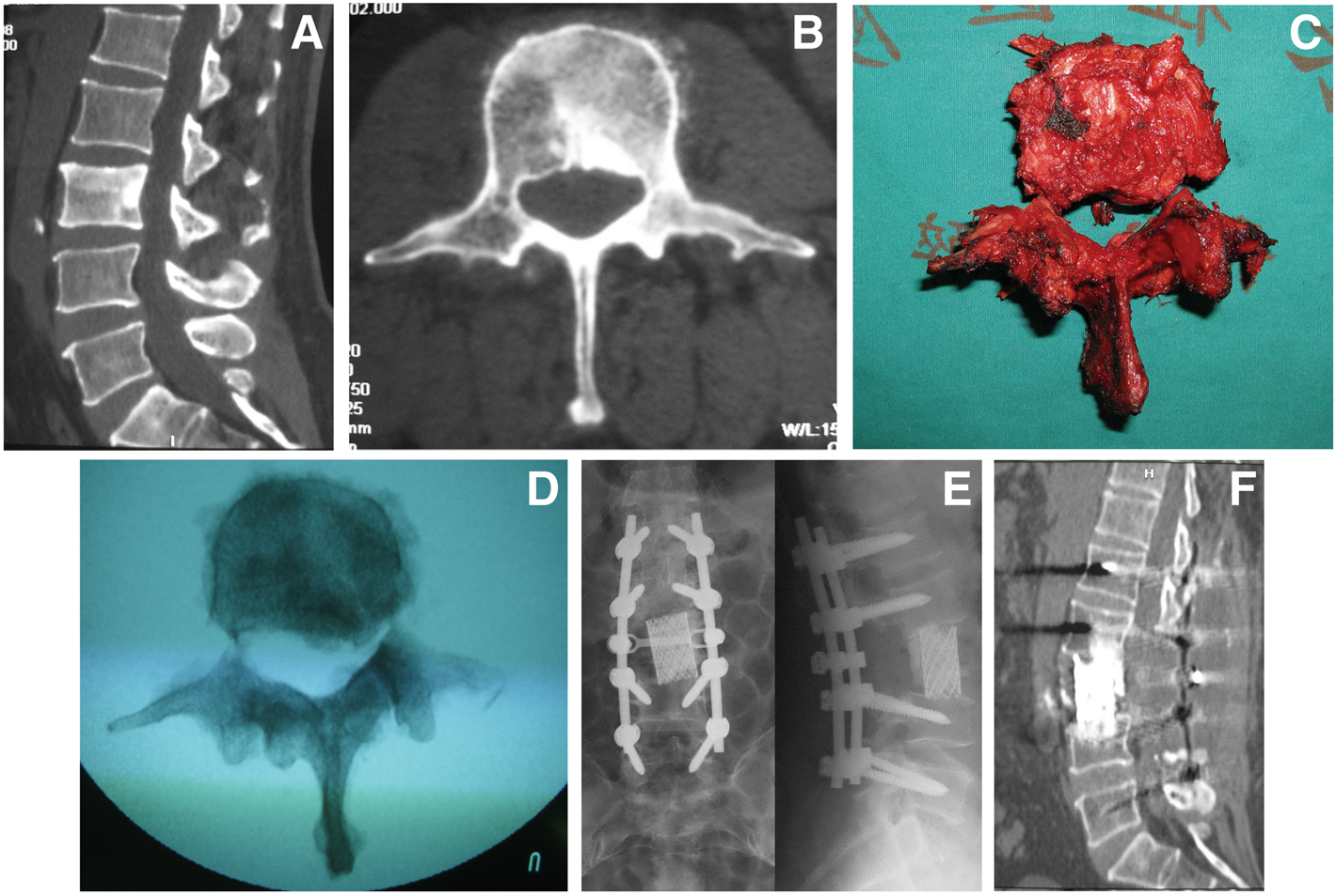
**Osteosarcoma affecting the L3 vertebra in a 55-year-old man (Case 11).** (**A, B**) Preoperative computed tomography (CT) scan showed the lesion within the L3 vertebra, right pedicle, and right lamina. Total *en bloc* spondylectomy, through a single posterior approach, was performed. (**C, D**) Extracted specimens included the posterior element and the anterior portion. (**E**) Titanium mesh filled with bone cement was used as anterior reconstruction, and a pedicle screw was applied in the posterior reconstruction. Shown is a postoperative radiogram demonstrating the correct alignment of instrumentation. (**F**) A CT study at 18 months after surgery demonstrated no relapse.

For the 16 patients with a tumor in their mobile spine, two different protocols of surgical treatment were chosen, based on the location and extension of the tumor according to the WBB and Enneking systems (Table 1). Seven patients were given total *en bloc* spondylectomy (TES; Figure 2): three of these patients had lesions in their thoracic spine and four had lesions in their lumbar spine. Total *en bloc* spondylectomy was performed on six patients through a single posterior approach and, on one patient with an osteosarcoma of the L5, through both an anterior and posterior approach.

**Table 1:** Clinical data for 16 cases of spinal osteosarcoma

**Number**	**Sex**	**Age**	**Site**	**Preoperative Frankel score**	**Ennking**	**WBB**	**Preoperative chemotherapy**	**Resection**	**Margin**	**Postoperative chemotherapy**	**Radiotherapy**	**Local recurrence**	**Metastasis**	**Follow-up**	**Postoperative Frankel score**	**Dead**
1	F	22	C1	E	IIA	3-6/A-C	Yes	Sagittal	Intralesional	Yes	No	Yes	Yes	44	E	No
2	M	41	C2-3	C	IIB	2-11/A-D	Yes	TPS	Intralesional	Yes	No	Yes	Yes	75	E	Yes
3	M	58	C3	C	IIB	2-9/A-D	Yes	TPS	Intralesional	Yes	Yes	No	Yes	39	D	No
4	M	50	C6	B	IIB	4-8/A-D	No	Vertebrectomy	Intralesional	Yes	Yes	Yes	Yes	50	D	Yes
5	M	27	C7	E	IIB	3-7/A-D	Yes	TPS	Intralesional	Yes	Yes	No	No	53	E	No
6	F	40	C7	D	IIB	2-9/A-D	Yes	TPS	Intralesional	Yes	Yes	No	No	36	D	No
7	M	30	T2	C	IIB	1-3,6-12/A-D	Yes	TPS	Intralesional	Yes	Yes	No	No	50	D	No
8	M	49	T4	E	IIB	6-10/A-D	Yes	TES	Marginal	Yes	Yes	No	No	35	E	No
9	M	28	T5	E	IIB	4-12/A-D	Yes	TES	Contaminated	Yes	Yes	Yes	Yes	40	E	Yes
10	F	49	T7	D	IIB	8-11/A-D	Yes	TES	Marginal	Yes	Yes	No	No	24	E	No
11	F	19	T7-9	B	IIB	3-12/A-D	No	TPS	Intralesional	Yes	Yes	Yes	Yes	10	C	Yes
12	F	17	T10-12	C	IIB	2-11/A-D	Yes	TPS	Intralesional	Yes	Yes	Yes	Yes	25	D	Yes
13	M	15	L1	E	IIIA	4-6/B-C	Yes	TES	Wide	Yes	Yes	No	Yes	37	E	No
14	F	48	L2	D	IIB	4-8/A-D	Yes	TES	Marginal	Yes	Yes	No	Yes	40	E	No
15	M	55	L3	E	IIA	7-10/B-C	Yes	TES	Marginal	Yes	Yes	No	No	71	E	No
16	M	46	L5	E	IIB	3-10/A-C	Yes	TES	Marginal	Yes	Yes	No	No	50	E	No

*TES* total *en bloc* spondylectomy; *TPS* total piecemeal spondylectomy; *WBB* Weinstein-Boriani-Biagini.

Nine patients received piecemeal resection. Seven of the nine patients were given total piecemeal spondylectomy. Within this group, four patients who had cervical lesions received a one-stage anteroposterior approach, while three patients who had thoracic lesions received a posterior approach alone. An eighth patient, with tumors at her C1 lateral mass, underwent sagittal resection through a one-stage anterior and posterior combined approach, while the final patient, with tumor at C6, underwent vertebrectomy through an anterior approach. During surgery, because of arterial involvement, one patient was given unilateral ligation of the vertebral artery. Additionally, nerve root ligation was performed in three patients, as planned, before surgery. All tumors and margins underwent histological examination after surgical excision.

Fourteen patients were given preoperative combination chemotherapy, including one course of cisplatin, doxorubicin, and high-dose methotrexate. All patients received postoperative chemotherapy. In addition, 14 patients received postoperative photon radiotherapy, with doses ranging from 35 to 65 Gy.

### Reconstruction

Reconstruction methods were chosen according to the surgical procedure chosen and with the aim of maintaining spinal stability and balance. Occipitocervical fusion by bone graft combined with occipitocervical internal fixation was used for the lesions at the upper cervical spine. The vertebral body was replaced by an anterior titanium plate and titanium mesh filled with bone cement. Posterior internal fixation with a pedicle screw system was given to the patients who had sacrificed the zygapophyseal joint, particularly the junctional regions.

## Consent

Written informed consent was obtained from the patient for publication of this report and any accompanying images.

Written informed consent was obtained from the patient’s parents for publication of this report and any accompanying images.

## Results

### Overall clinical findings

Sixteen patients with a histologic diagnosis of osteosarcoma and surgical treatment were observed from 10 to 75 months of follow-up (average: 42.4 months). The date of follow-up evaluations was obtained from office visits and telephone interviews. Three months following their surgical procedure, most patients’ pain eased or gradually disappeared. There was also a significant improvement in the patients’ neurologic deficits following removal of their tumor. Eight patients had an improved neurologic status, with Frankel grade scores of 1 to 2, at three months after surgery.

Overall, six of the sixteen patients (37.5%) experienced local recurrence of the tumor during the follow-up period while nine of the sixteen patients (56.3%) developed metastases. One patient from the metastatic group, with an osteosarcoma in his left inferior femur and left upper end of the humerus, despite receiving a wide tumor resection, later developed a solitary spinal metastasis. The other eight patients developed metastasis after surgery. Five of the sixteen patients (31.3%), with both local recurrence and tumor metastasis, died of their disease (Table 1).

### Surgical procedure and outcomes

Nine patients received piecemeal resections; there were seven instances of total piecemeal spondylectomy, one instance of sagittal resection, and one instance of vertebrectomy. All resections were later found to be intralesional. Five of these nine patients experienced a relapse from 4 to 34 months post-surgery (average: 18.8 months), and five patients developed metastases. Four of these patients died because of infection of the lung, caused by pulmonary metastasis and multiple organ failure (Table 1).

Total *en bloc* spondylectomy was performed in the remaining seven cases (Figure 2), all for lesions within the thoracolumbar spine. Histologic assessments of the surgical margins showed a wide margin in one patient, a marginal zone in five patients, and a wide contaminated margin in one patient. Three of these patients had metastases to the lung and bone and, of these, one who received tumor resection with a wide contaminated margin had a relapse at 28 months and eventually died (Table 1).

None of the six patients who received surgical resection with a wide or marginal margin developed local recurrence or died of the disease. However, of the ten patients who obtained intralesional or wide contaminated resections, six patients (60%) relapsed and five (50%) died of their disease (Table 1).

### Complications

One patient developed intraoperative anaphylactic shock because of a blood transfusion. Prompt recognition of this problem led to the patient’s safe recovery. The unilateral vertebral artery was ligated in one case during the operation, with no postoperative complication. The three patients whose nerve root was ligated and cut complained of a dull, sustained pain around the wound, but tolerated the procedure without the need for narcotics. Three patients had transient cerebrospinal fluid leak from the wound after surgery and two of these three patients developed subsequent infection (*Staphylococcus aureus* and *S. epidermidis*) of the cerebrospinal fluid. The symptoms for these patients included hyperpyrexia, headache, and dehiscence of the incision. The patients were given standard antibiotic therapy, debridement, and wound revision. Finally, the symptoms disappeared and the incision healed.

## Discussion

The spine is rarely affected by osteosarcoma. Primary spinal osteosarcoma accounts for 0.85–3.0% of all osteosarcomas [8] and 3.6% to 14.5% of primary spinal tumors [9,10]. While extremity osteosarcoma is mainly seen in children and adolescents, spinal osteosarcoma tends to occur in an older age group [11]. In our group, thirteen of 16 cases (81.3%) were older than 20 years in our study, the average age being 37.1 years.

Treatment of osteosarcoma is described in various studies [4,8,11-14]. Surgical resection of the osteosarcoma is a critical part of the treatment regime, in addition to adjuvant chemotherapy and radiation. Because of the relatively complicated anatomic constraints of the spine and its complex blood supply, it is necessary to develop an optimal surgical plan before the procedure, aided by the WBB and Enneking systems. The WBB staging system involves dividing the spine segments with the tumor into 12 radiating zones (numbered 1 to 12 in a clockwise fashion). This classification also takes into account the transverse plane and five layers (A to E, from the paravertebral extraosseous region to the area of dural involvement) of the spine segment. The Enneking system, on the other hand, includes three stages. Stages I and II are based on the surgical grade of the tumor without metastasis. Stage III represents the tumor with metastasis. Each stage is further divided into two subcategories (A, B) based on the local extent of tumor.

Enneking *et al.*[15] proposed the concept of staging based on tumor compartmentalization and anatomic barriers and Tomita *et al.*[16] applied this concept to the spine. They concluded that, in the spine, one vertebra could be regarded as a single oncological compartment and its surrounding tissues (for example, ligaments, periosteum, and cartilage) could be regarded as barriers. Using this approach, the authors developed the TES technique, wherein the whole vertebra, both body and lamina, are resected as one compartment. Conversely, in total piecemeal spondylectomy, the vertebra is resected in a piecemeal fashion and further tissue is subsequently removed to achieve a wide margin. Total piecemeal spondylectomy is an appropriate choice for those patients with osteosarcoma in the spine who are not candidates for *en bloc* resection. It is important to know that an *en bloc* resection occurs in an excision fashion that is not synonymous with a wide or marginal margin, whereas a piecemeal resection is synonymous with the term ‘intralesional resection’.

There are only a few smaller studies describing outcomes after TES. Liljenqvist *et al.*[17] reported a study of four patients with spinal osteosarcoma who underwent TES with wide or marginal margins. In the 75-month follow-up period, two were alive with evidence of disease while the other two had no evidence of disease. Abe *et al.*[18] reviewed two patients with osteosarcoma of the spine who received TES. One patient obtained a wide margin and had no relapse. The second obtained an intralesional margin and experienced local recurrence. These studies suggest that TES with a wide margin is effective in controlling local recurrence. In addition, the effectiveness of TES (with a wide or marginal margin) has already been shown, in terms of local control and improved long-term prognosis for osteosarcoma of the thoracolumbar spine [4]. However, owing to the complex anatomy of the spine, the use of TES for treatment of osteosarcoma in the cervical spine has not been previously reported. Seven cases of our series underwent TES treatment. Of these, none of the six cases who obtained a wide margin had local recurrence. The only patient who relapsed, and ultimately died of the disease, obtained a wide contaminated margin. We also analyzed cases receiving piecemeal resection procedures. In our study, five of the nine patients (55.5%) who received piecemeal resection developed local recurrence, with four of these patients (44.4%) eventually succumbing to their disease. These results indicate the important role of TES (performed with wide or marginal margins) for the successful treatment of spinal osteosarcoma. Moreover, patients who received TES had a better prognosis than those patients who received piecemeal spondylectomy.

Of the nine patients receiving piecemeal resection, seven cases (four cervical lesions and three thoracic lesions) underwent total piecemeal spondylectomy through the one-stage anteroposterior approach or a posterior approach alone. Three of these seven patients (42.9%) developed local recurrence of the tumor. Of the remaining two patients receiving piecemeal resection, one received sagittal excision and another received a vertebrectomy. Both of these patients experienced tumor recurrence. Because of the limited number of patients, these results are not conclusive, but a trend was observed. Patients who received total piecemeal spondylectomy had a better prognosis. Xiao *et al.*[19] presented a study of 39 patients with cervical bone tumors, who received total piecemeal spondylectomy. The author concluded that total piecemeal spondylectomy, with both an anterior and posterior approach, can reduce local recurrence. Sundaresan *et al.*[20] reported that the use of total piecemeal spondylectomy prolonged the average survival time of patients with thoracolumbar tumors.

While *en bloc* resection is the best choice, surgical planning should include the possible sacrifice of important structures to maximize the success of oncological surgery. In some cases, the nerve root needs to be sacrificed in surgery, although the goal is always to preserve the cervical and lumbar nerve roots, if possible. Except for at T1, the thoracic nerve roots can be ligated. In the present series, three thoracic nerve roots were sacrificed because the nerve root was behind and obstructed the vertebral body removal, from an anterior to posterior approach. Nerve removal did not cause serious neurologic deficits after surgery. The vertebral artery is unique to the cervical spine and mandates the surgical plan. A preoperative angiographic study of vertebral arteries should be given, to identify the relationship between the arteries and the tumor. The vertebral artery can be ligated only when the patient tolerates the occlusion test. During total spondylectomy of the cervical tumor, one of our patients experienced unilateral artery ligation, which did not cause postoperative neurological symptoms.

It is well known that osteosarcoma is sensitive to chemotherapy. The standard treatment for osteosarcoma of the extremities is a combination of wide surgical excision and chemotherapy. Neoadjuvant chemotherapy can eliminate systemic micrometastases and improve prognosis. In our study, 14 patients were given preoperative chemotherapy and all patients received postoperative chemotherapy. The two patients who were not given preoperative chemotherapy had local recurrences after surgery and died of the disease.

In the past, radiation therapy has not been widely utilized for the treatment of osteosarcoma, as it is usually considered to be radioresistant. However, with recent advances in radiation therapy, there are several reports documenting tumor control in patients with incompletely resected tumors or who are not candidates for surgery. In our study, 14 patients received radiotherapy after their operation. The two patients who refused postoperative radiotherapy experienced local relapse, although because of the small sample size, we cannot draw a statistical conclusion from these data. We still presume that radiotherapy could be regarded as a beneficial supplement after surgery.

Osteosarcoma of the spine has a poor prognosis. Mukherjee *et al.*[12] reviewed the National Cancer Institute’s Surveillance, Epidemiology, and End Results (SEER) database for 1,892 patients with spinal neoplasms. Their series included 430 patients with osteosarcoma who received surgical treatment and radiotherapy: 78% of these patients died during their SEER follow-up period and 28% developed metastasis. In agreement with this study, Shives *et al.*[13] documented 27 cases with spinal osteosarcoma and found that 26 (96.3%) of the patients had died of the disease from 1 to 18 months after surgery. Ozaki *et al*. [14] reviewed the results of 22 patients with osteosarcoma of the spine. All 22 patients were given chemotherapy, 12 patients underwent surgery, and 8 patients received irradiation. Sixty-four percent of these patients died of disease within two years. Furthermore, their study found a correlation between intralesional or no-tumor surgeries and poorer survival.

In our series, after treatment, 37.5% of the cases experienced local recurrence, 56.3% had metastases, and 31.3% died during the 72-month follow-up, at an average of 42.2 months post-surgery (Table 2). The results of our study also demonstrated that these patients experienced a high recrudesce rate, high distant metastasis rate, and high mortality rates.

**Table 2:** Surgical treatment and outcomes of 16 cases of osteosarcoma of the mobile spine

**Treatment (type)**	**Number**	**Local recurrence (%)**	**Metastasis (%)**	**Dead (%)**
TES + wide or marginal	6	0	2 (33.3%)	0
TES + contaminated	1	1	1	1
Piecemeal	9	5 (55.6%)	6 (66.7%)	4 (44.4%)
**Total**	**16**	**6 (37.5%)**	**9 (56.3%)**	**5 (31.3%)**

*TES* total *en bloc* spondylectomy.

## Conclusions

Osteosarcomas commonly originate in the extremities, and only rarely in the spine. Osteosarcoma of the spine has a high rate of recurrence, metastasis, and mortality. Our study supports the conclusion that patients with osteosarcoma in the cervical or thoracolumbar spine should be treated with a combination of TES, with a wide or marginal margin, and chemotherapy. Radiotherapy may also be of benefit in selected patients. Because of the complicated anatomic structure of the spine, TES is technically challenging and not indicated for some selected patients with spinal tumors. In these instances, total piecemeal spondylectomy is a good alternative for a wide resection of the whole spinal tumor compartment and is an appropriate choice for osteosarcoma in the mobile spine.

## Abbreviations

CT: Computed tomography; MRI: Magnetic resonance imaging; SEER: Surveillance, epidemiology, and end results; TES: Total *en bloc* spondylectomy; TPS: Total partial spondylectomy; WBB: Weinstein-Boriani-Biagini.

## Competing interests

The authors declare that they have no competing interests.

## Authors’ contributions

Feng D, Yang X, Liu T,Xiao J conceived and designed the study. Wu Z, Huang Q, Ma J, Huang W, Zheng W, Cui Z, Xu H, Teng Y collected data. Feng D and Xiao J wrote the paper. All authors read and approved the final manuscript.
